# Breast tumor response to ultrasound mediated excitation of microbubbles and radiation therapy *in vivo*

**DOI:** 10.18632/oncoscience.299

**Published:** 2016-03-24

**Authors:** Priscilla Lai, Christine Tarapacki, William T. Tran, Ahmed El Kaffas, Justin Lee, Clinton Hupple, Sarah Iradji, Anoja Giles, Azza Al-Mahrouki, Gregory J. Czarnota

**Affiliations:** ^1^ Radiation Oncology, Sunnybrook Health Sciences Centre, Toronto, Ontario, Canada; ^2^ Department of Radiation Oncology, University of Toronto, Toronto, Ontario, Canada; ^3^ Imaging Research, Sunnybrook Health Sciences Centre, Toronto, Ontario, Canada; ^4^ Department of Medical Biophysics, University of Toronto, Toronto, Ontario, Canada

**Keywords:** Microbubbles, high frequency ultrasound, microvasculature, radiation, breast neoplasm

## Abstract

Acoustically stimulated microbubbles have been demonstrated to perturb endothelial cells of the vasculature resulting in biological effects. In the present study, vascular and tumor response to ultrasound-stimulated microbubble and radiation treatment was investigated *in vivo* to identify effects on the blood vessel endothelium. Mice bearing breast cancer tumors (MDA-MB-231) were exposed to ultrasound after intravenous injection of microbubbles at different concentrations, and radiation at different doses (0, 2, and 8 Gy). Mice were sacrificed 12 and 24 hours after treatment for histopathological analysis. Tumor growth delay was assessed for up to 28 days after treatment. The results demonstrated additive antitumor and antivascular effects when ultrasound stimulated microbubbles were combined with radiation. Results indicated tumor cell apoptosis, vascular leakage, a decrease in tumor vasculature, a delay in tumor growth and an overall tumor disruption. When coupled with radiation, ultrasound-stimulated microbubbles elicited synergistic anti-tumor and antivascular effects by acting as a radioenhancing agent in breast tumor blood vessels. The present study demonstrates ultrasound driven microbubbles as a novel form of targeted antiangiogenic therapy in a breast cancer xenograft model that can potentiate additive effects to radiation *in vivo*.

## INTRODUCTION

The introduction of microbubbles as a contrast agent in ultrasound imaging has led to improvements in the quality of diagnostic imaging. Extensive research has been conducted to develop stable, biocompatible, microbubble-based contrast agents that are safe for clinical imaging applications. Microbubbles reach a steady state intravenously and persist in the blood for several minutes [1]. The high acoustic impedance of microbubble gas compared to the surrounding tissues creates significant contrast and permits perfusion-based imaging using ultrasound [2, 3]. Regulating the acoustic exposure parameters can lead to varying microbubble effects, such as oscillations and cavitations that are detectable at harmonic frequencies [3–5].

Recent studies have investigated the potential therapeutic benefits of acoustically stimulated microbubbles in biological systems. These studies have reported increased vascular permeability, decreased vascular integrity, the creation of vascular-based lesions, hemorrhaging, and endothelial cell death depending on ultrasound pressures and frequencies used [4, 6–9]. *In vivo* studies have demonstrated the use of acoustically stimulated microbubbles to re-open acutely thrombosed vessels using either platelet-targeted or lipid-encapsulated microbubbles [10–12]. The resulting biological effects on the surrounding cells and vasculature indicate a potential for a range of therapeutic effects of ultrasound- stimulated microbubbles spanning from drug delivery, anti-angiogenic effects, and an increased sensitivity to anticancer treatments [13–16]. In addition, microbubbles are being investigated for their potential role in drug and gene delivery through sonoporation [17–19]. There is evidence to suggest that ultrasound-driven microbubbles increase the permeability of the cell plasma membrane, caused by stable and inertial cavitation [17–22]. This principle has led to recent findings which demonstrate a transient opening of the blood brain barrier as a result of ultrasound-microbubble exposure [23–26] and have raised more questions about its potential applications for therapy.

Recent research in radiation oncology has focused on disrupting endothelial cells of the tumor vasculature by using acoustically stimulated microbubbles. The effects have been shown to potentiate radiation damage through concomitant cell death signalling initiated from damaged endothelium and tumor cells [8, 9, 27, 28]. There is evidence to suggest that shear stress to the plasma membrane of endothelial cells may cause ceramide-mediated ASMase signalling, which drive apoptotic cell death [8, 27–29]. When combined with radiation, cascading cell death signals from both the vasculature and tumor cells are believed to cause additive tumor damage. Our previous work has shown that combined radiation and ultrasound-mediated microbubbles cause a decrease in vascular perfusion, increased tumor cell death, and delays in tumor growth with improved survival in pre-clinical prostate and bladder models [8, 9]. The therapeutic yield in those previous treatment models were also dependent on the time between the two treatments [22], microbubble concentration and time of ultrasound exposure [8, 22, 27, 29]. Acoustic parameters such as the peak negative pressure and frequency were also shown to affect tumor response in combination treatments [3, 14].

In the present study, we aim to build on our previous studies and examine the effects of combined radiation and microbubble treatment in a breast cancer model. The motivation for this current work is based on previous results suggesting differences in endothelial cell membrane permeability from ultrasound-mediated microbubbles among varying tumor models [4, 7, 30]. We hypothesize that these differences may also affect combined-treatment response phenotypes in breast cancer. Therefore, the present study investigates a highly vascularized breast tumor type, in response to ultrasound- driven microbubbles and radiation. Tumor response was studied at two time intervals following treatment (12 hours and 24 hours) and tumors were also monitored for growth delay in response to therapy.

## RESULTS

**Figure 1:**
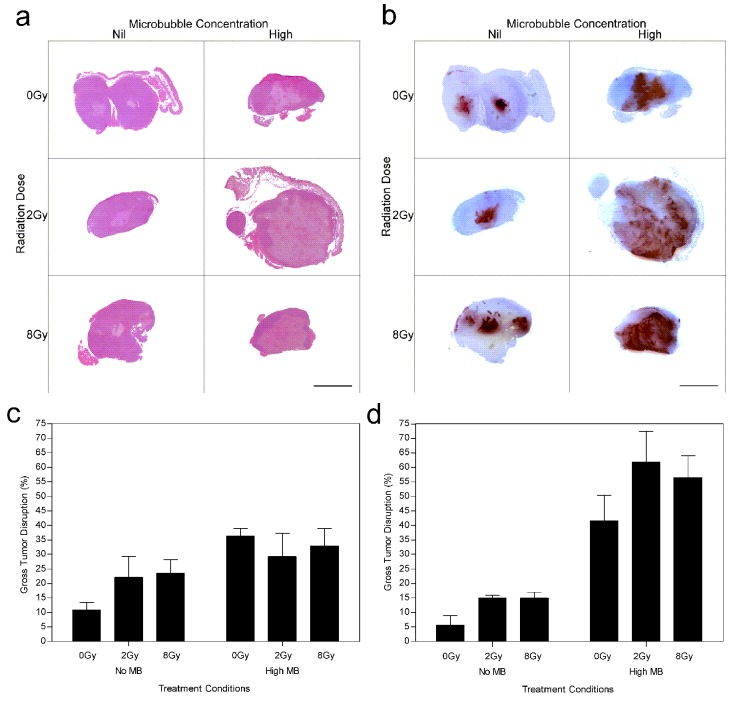
Gross tumor histopathology of MDA-MB-231 human breast tumor xenografts after ultrasound-microbubble and radiation treatments Low magnification (a) H&E and (b) ISEL stained histological sections of treated tumors (24 h cohort). Summary of percent gross tumor disruption per treatment group determined from ISEL stained histology sections for the (c) 12 h and (d) 24 h cohorts. Treatment conditions include combinations of nil (0 %) and high (3 % v/v) microbubble concentrations with 0 Gy, 2 Gy and 8 Gy radiation dose. The scale bar denotes 5 mm.

Results revealed negligible cell death when 2 Gy radiation was given alone compared to untreated animal tumors which demonstrated central tumor necrosis. Increased cell death was predominantly visible at 8 Gy and ultrasound-microbubble treated conditions. Histological analysis was used to assess the degree of tumor cell death (Figure 1). H&E and ISEL demonstrated gross tumor response 24 h after treatment (Figure 1a and 1b). Results indicated that there was increased tumor response when higher radiation doses were administered at 24 h (Figure 1d) while showing negligible differences at 12 h (Figure 1c). Gross tumor disruption was 15 ± 4% for 2 Gy and increased to 29 ± 4% for an 8 Gy radiation dose at 24 h. Exposure to ultrasound-driven microbubbles alone caused cell death when compared to the control, increasing cell death from 10 ± 2% to 26 ± 5% (*P*_mc_ < 0.05). Combination treatments with microbubble-ultrasound and radiation resulted in significant synergistic effects in cell death and were predominantly observed in the tumor's central region (Figure 1b).

Tumor response after 12 h (Figure 1c) and 24 h (Figure 1d) was quantified using ISEL stained histology, revealing enhanced cell death when ultrasound-driven microbubbles were combined with radiation. Data indicated that cell death increased from 12 to 24 hours after ultrasound-microbubble treatment. For the 12 h cohort the average increase in tumor death from combining ultrasound-microbubble therapy with radiation was 1.3 (± 0.4) times for 2 Gy dose, and 1.4 (± 0.4) times for 8 Gy dose (*P*_mc_ < 0.01). Results demonstrated an increase in cell death after 24 h for ultrasound-microbubble treated conditions. There was a 3.4 (± 1.0) fold increase and 2.3 (± 0.4) fold increase for the 2 Gy and 8 Gy treatment groups, respectively (*P*_mc_ < 0.0001) at the 24 h time point. Tumors from the control group demonstrated inherent (baseline) tumor death measured at 6-12% consistently throughout the study.

**Figure 2:**
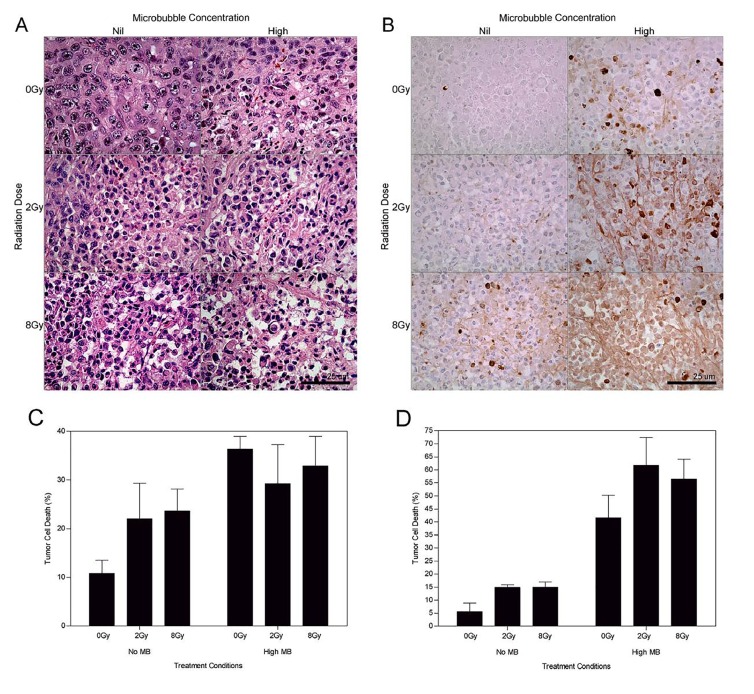
High magnification (a) H&E and (b) ISEL stained histological sections of treated MDA-MB-231 tumors (24 h cohort) Summary of the apoptotic index per treatment group determined from ISEL stained histology sections for the (c) 12 h and (d) 24 h studies. Treatment conditions include combinations of nil (0 %) and high (3 % v/v) microbubble concentrations with 0 Gy, 2 Gy and 8 Gy radiation dose. The scale bar denotes 50 μm. Obtained at magnification = 400×.

High magnification microscopy was used to visualize treatment effects on cellular morphology (Figure 2). Haematoxylin and eosin stained assessment of the 24 hour treatment group (Figure 2a) indicated tumor cell disruption when ultrasound-stimulated microbubbles were used in conjunction with radiation. Areas of retraction artefact indicative of apoptosis were found when the 2 Gy and 8 Gy radiation doses were combined with ultrasound-stimulated microbubbles.

ISEL stained samples revealed an increase in detectable apoptotic cells (Figure 2b). Samples showed sparsely organized cells, cell heterogeneity, nuclear condensation and fragmentation. Prominent apoptotic cell death and cellular disruption was evident with the 2 Gy dose combined with ultrasound-stimulated microbubbles. For the 12 h cohort the average increase in apoptosis for treatment groups with radiation and ultrasound-microbubbles was 1.5 (± 0.2) times for 2 Gy dose, and 1.4 (± 0.2) times for 8 Gy dose (*P*_mc_ < 0.01) (Figure 2c). Increases in the radiation dose also resulted in an increase in the apoptotic index by 1.8 (±0.2) times for the 2 Gy treatment groups, and 2.7 (±0.2) for the 8 Gy treatment groups after 12 hours (*P*_r_ < 0.0001). At 24 hours following treatment, tumors showed an increase in detectable apoptosis when compared to the 0 Gy group. We observed an increase of 7 (±3) times for the 0 Gy treatment groups, 4.1 (±0.6) times for the 2 Gy treatment groups, and 3.9 (±0.8) for the 8 Gy treatment groups (*P*_r_ < 0.0001) (Figure 2d).

**Figure 3:**
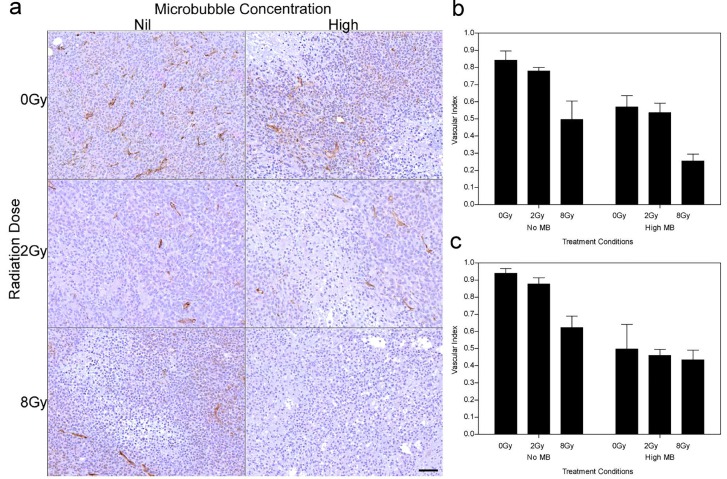
CD31 stained tumors showing microvascular disruption after treatment (a) High magnification stained histological sections of treated tumors (24 h cohort). Vascular indices determined from the CD31 results, for each treatment condition, are summarized for the (b) 12 h and (c) 24 h studies. The scale bar denotes 50μm. Obtained at magnification = 80×.

Vascular disruption was assessed using CD31 immunohistochemistry (Figure 3). Analysis of the tumor vasculature revealed that 2 Gy radiation and high ultrasound-stimulated microbubble treatment resulted in a reduced vascular index of 0.63 (± 0.09) times for the 12 h group and 0.58 (± 0.09) times for the 24 h group (*P*_mc_ < 0.001). Treatments with 8 Gy radiation also resulted in a significantly lower vascular index compared to the 0 Gy and 2 Gy treatment groups. Vascular indices of the combined treatment were 0.5 (± 0.1) times and 0.6 (± 0.1) times lower than radiation alone for 0 Gy and 2 Gy doses respectively for the 12 h cohort (*P*_r_ < 0.001).

**Figure 4:**
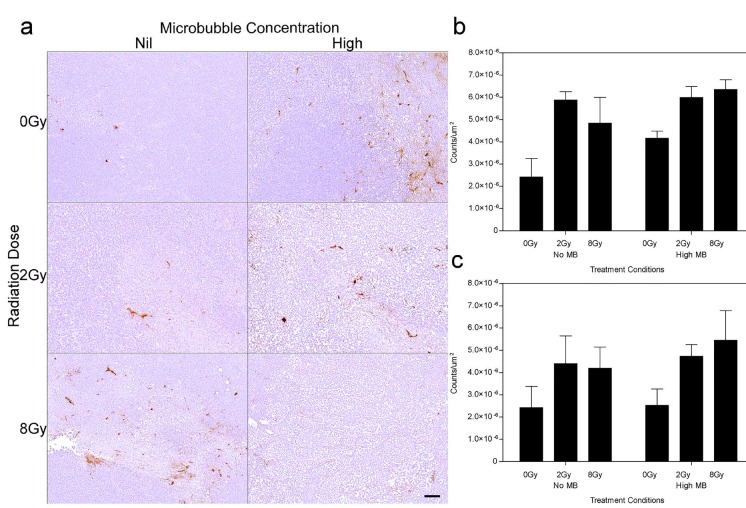
Factor VIII stained xenografts showing vascular leakage after tumor treatment (a) High magnification stained histological sections of treated tumors (24 h cohort). Vascular damage determined from the Factor VIII results, for each treatment condition, are summarized for the (b) 12 h and (c) 24 h studies, and expressed in counts/um2. The scale bar denotes 50 μm. Obtained at magnification = 100×.

Vascular leakage was assessed using Factor VIII staining (Figure 4). The results indicate that exposure of xenografted tumors to ultrasound-stimulated microbubbles alone resulted in vascular leakage when combined with radiation at 12 hours and 24 hours (Figure 4b, 4c). Furthermore, the difference in observed vascular leakage between 2 Gy and 8 Gy treatments was not significant. A decrease in vascular leakage of 13-39% was observed within treatment groups between the 12 h (Figure 4b) and 24 h (Figure 4c) cohorts.

**Figure 5:**
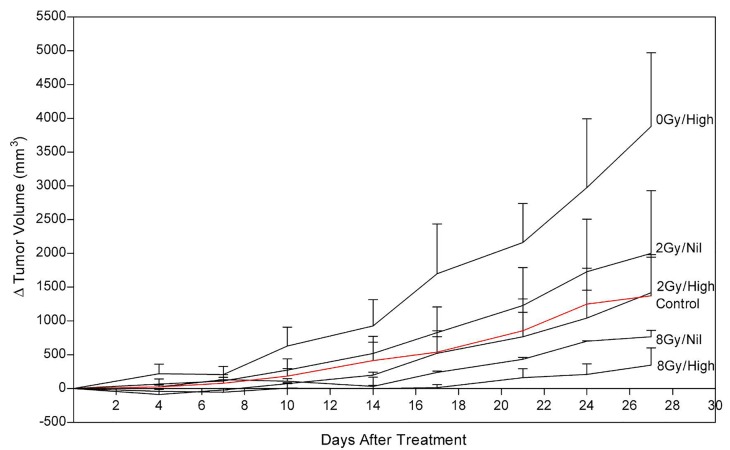
Tumor growth delay per treatment group (n=3), expressed as a change in tumor volume (mm3), and monitored over a total duration of 28 days Times between consecutive measurements were 3-4 days.

Tumor size and growth delay after one treatment session was assessed up to 28 days post treatment (Figure 5). Tumors treated with combined ultrasound- stimulated microbubbles and 2 Gy radiation exhibited a delay in growth compared to the control. The ultrasound- stimulated microbubble treatment alone (0Gy/High) initiated a delay in tumor growth, and then relapsed into recovery. After 27 days, tumors treated with ultrasound- stimulated microbubble treatment combined with 2 Gy or 8 Gy recovered. However, these tumors had become 60% and 30% smaller than the control group, respectively. Tumor growth was delayed for a mean of 17 days in the ultrasound-stimulated therapy combined with 8 Gy (8Gy/High). Treatments with combined radiation, ultrasound and microbubble therapy demonstrated superior growth inhibition when compared to radiation only. Treatment with ultrasound-stimulated microbubbles alone resulted in tumor rebound and had a 2.8 fold-increase in size compared to the control group at day 27. The treatment with 2 Gy alone (2Gy/Nil) also resulted in greater growth in comparison to the untreated group (approximately a 1.5 fold-increase at 27 days).

**Figure 6:**
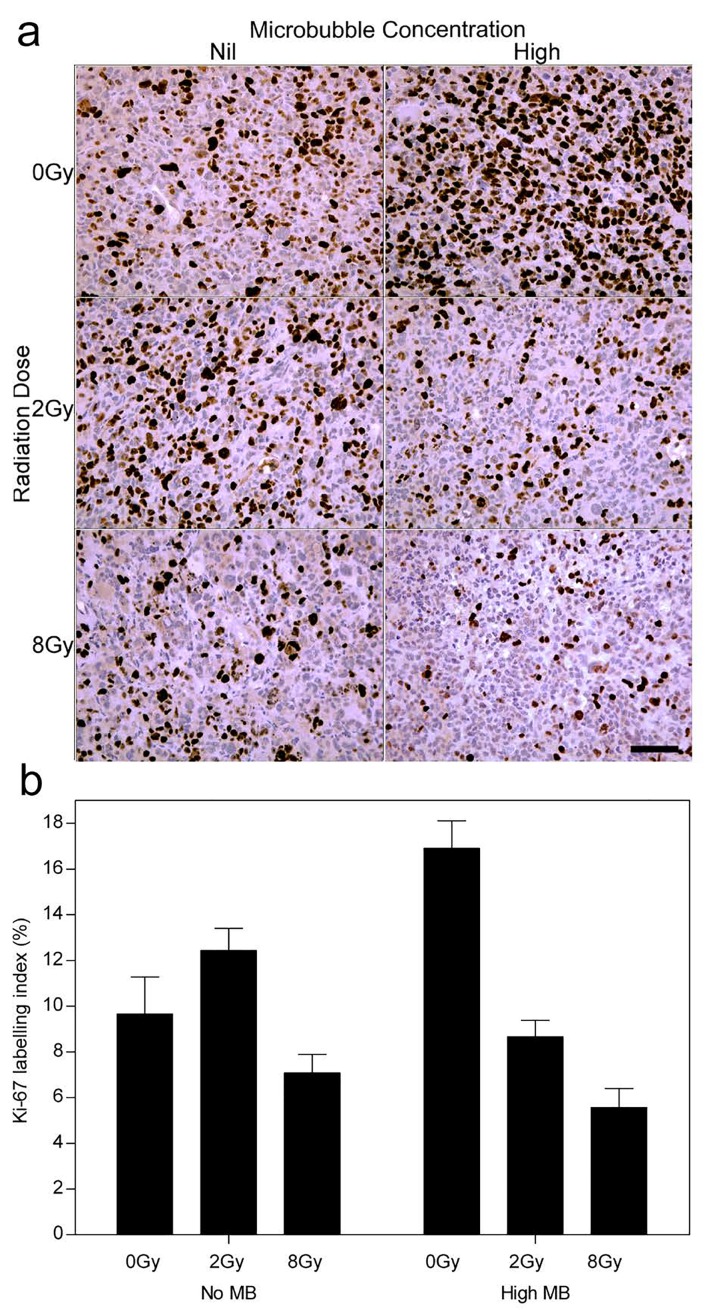
Ki-67 stained histological sections showing the growth fraction of the cell population (a) High magnification images from the long term cohort (< 28 days). (b) Ki-67 labelling index (%) summary from tumor growth delay endpoint animals. The scale bar denotes 50 μm. Obtained at magnification = 200×.

Ki-67 analysis was conducted to test for the proliferative fraction (Figure 6). Control tumors showed an index of 9 (± 2)%. Tumors treated with ultrasound- stimulated treatments alone (0 Gy, High MB) and 2 Gy alone yielded an index of 17 (± 3)% and 13 (± 3)% respectively (Figure 6b). Tumors treated with combined ultrasound-stimulated microbubbles and 2 Gy or 8 Gy showed a decrease in Ki-67 labelling indices to approximately 9 (± 1)% and 6 (± 1)%, respectively (*P*_i_ < 0.001).

## DISCUSSION

Ultrasound-stimulated microbubbles were used in this study to induce vascular disruption in tumors. The results from this present study demonstrate that ultrasound-driven microbubbles can serve as a potent modality in disrupting tumor vasculature and can potentiate the effects of radiation in a breast cancer model. Recent studies have investigated endothelial cell responses to microbubble-based ultrasound contrast agents [8, 9, 31] and radiotherapy [9, 32]. Those studies demonstrated a mechanism for the enhancement of radiation responses consisting of endothelial cell perturbation leading to the activation of gene expression pathways, which are stimulated with radiation (discussed further below). This leads to endothelial cell apoptosis and vessel damage leading to vascular collapse. Tumor cell death is secondary to this blood flow shutdown leading to up to 60% tumor cell death after a single 2 Gy radiation dose when combined with ultrasound-stimulated microbubble exposure *a priori*.

Additionally, *in vitro* studies have demonstrated that ultrasound-stimulated microbubbles can induce physical changes in the biological integrity and morphology of endothelial cells which can subsequently initiate cell signals associated with cell stress. These same pathways are stimulated by radiation responses in endothelial cells. Other studies conducted *in vivo* in murine and porcine models have supported findings that endothelial cells are perturbed from microbubble-ultrasound exposure within the vasculature [33, 34]. Similar results have been demonstrated in further studies investigating alterations to auricular vessels exposed to microbubble-based contrast agents and focussed ultrasound [6, 7]. Localized vascular injuries, such as vascular wall damage and haemorrhaging leading to necrosis, were also observed in the brains of rabbits intravenously injected with a microbubble contrast agent and insonified with ultrasound [35]. Shear stress caused by violent oscillations of microbubbles in the kidneys may lead to intratubular obstruction [36]. It would be reasonable to speculate that the tumor response profile observed in the study here, such as decreased vascularity and increased vessel leakiness, are a result of some of these mechanisms. Other studies have addressed the ability of ultrasound-stimulated microbubbles to accelerate tissue heating in murine kidneys [37] and induce apoptosis in malignant human lymphoblast cells [38].

When combined with radiation, additive effects of ultrasound-stimulated microbubbles were observed here resulting in modifications to tumor biology and cell viability. These modifications were primarily detected as induced tumor cell apoptosis and vascular disruption. The effectiveness of these treatments has been previously demonstrated in other cancer lines [8, 9]. Similar to those other studies, it is suspected that endothelial cell injury from treatment may initiate cell signals that activate apoptosis [8, 9]. Cell signals such as those modulated by ceramide are particularly important in initiating apoptosis after injury to external stressors [22]. It has been previously demonstrated that ceramide production can increase by near 60% after treatment with ultrasound- stimulated microbubbles in breast cancer cells and can also increase by over 10% in endothelial cells [22]. In addition, there is evidence that demonstrates that ceramide is activated by modulators on the endothelial cell surface during radiation injury [32]. Therefore, these combined treatments are suspected in playing a role in synergistic signalling of the apoptosis pathway through combined ceramide induction. The results yield a biological response that requires a lower radiation dose in order to achieve relatively equivalent therapeutic outcomes to when higher doses of radiation are given alone. It is worthy to note the comparisons of this current study to the previous two studies that have examined prostate cancer response and bladder cancer response to adjuvant microbubble and radiation treatments *in vivo*. Firstly, it is important to test the feasibility of this technology as a treatment option for breast cancer as was done in the work here. Biological effective doses are dependent on tissue type and the current study demonstrates initial workings towards a framework to understand the effective dose required to achieve a therapeutic outcome similar to our previous studies in bladder and prostate cancer. The current study shows that treatment may be effective but an altered fractionation schedule of combined microbubble-radiation therapy may be required to achieve the same microbubble- ultrasound effective dose in other tissue types. Secondly, it is worthwhile to investigate the vascular response mechanisms to radiation and anti-vascular treatments in breast cancer. The current findings demonstrate that aggressive breast cancer models used may exhibit rapid vascular regeneration properties. This is evident in the vascular regrowth trend here which was not demonstrated to the same magnitude in other cancer models [8, 9]. Therefore, we suspect that the vascular architecture of this metastatic breast cancer may be more resistant to vascular disrupting agents and thus would require a modified dose to achieve equivalent responses. This may be attributed to recent findings by Nofiele *et al*. (2013) in which higher ceramide levels were associated with microbubble exposure in prostate cancer cells in comparison to breast cancer cell lines. Thirdly, the breast cancer data here in response to microbubble-ultrasound treatment serves as an initial framework in order to optimize the required acoustic parameters necessary to achieve desired biological responses in tumor microenvironment.

The ability of ultrasound-driven microbubbles to trigger the apoptotic pathway supports paradigms about radioenhancement by these agents and their effect on the vascular endothelium. This concept is founded from *in vivo* studies that have confirmed that ionizing radiation induces apoptosis in early endothelial cells [39]. It has been suggested in other studies that damage to microvasculature regulates the tumor cell response to radiation [40].

Assessment of tumor response at 12 h and 24 h using ISEL and H&E staining demonstrated that the use of ultrasound-driven microbubbles combined with radiation enhanced the tumor killing potential in comparison to delivering these modalities as a monotherapy. An increase in tumor disruption and cell death from 12 h to 24 h was not observed when microbubbles were absent. Endothelial markers such as CD31 and Factor VIII have been used to determine vascular density in other studies exploring the correlation between angiogenesis and metastasis of cancer [41, 42]. Evaluation of tumor vasculature using CD31 and Factor VIII immunohistochemical techniques have revealed decreases in vascular density and loss of vascular integrity from the combined ultrasound- driven microbubble and radiation therapy. In addition, our Ki-67 immunohistological assessment of tumors from treatment groups demonstrated that the proportion of actively dividing cells in tumors receiving combined radiation and activated microbubble therapy is lower than tumors receiving radiotherapy alone. Tumors treated with ultrasound-stimulated microbubbles alone demonstrated a tumor growth rate above the control growth rate. Proposed next steps would be to examine the microbubble dose and fractionation schemes required to limit the rapid recovery of breast cancer tumors from microbubble treatment alone.

## CONCLUSION

Tumor responses in a human breast cancer xenograft model are promising and suggest that microbubbles may be used as an adjuvant treatment modality with radiation in breast cancer. Additional benefits include a potential in dose reduction required to achieve similar radiobiological equivalent outcomes. Combination therapies would therefore potentially reduce adverse effects and maintain the effectiveness of treatment. Targeted therapy can be achieved by using focused ultrasound to eradicate disease at the disease site. The research in this study forms a basis for future research into mechanisms and optimizing the combination of the two treatments.

## MATERIALS AND METHODS

### Cell tissue culture

A human adenocarcinoma breast cell line (MDA- MB-231) from the American Type Culture Collections (ATCC, MD, USA) was cultured in RPMI-1640 medium supplemented with 5% penicillin/streptomycin antibiotic and 10% fetal bovine serum. The cells were incubated at 37°C in 5% CO_2_ and allowed to reach confluence. In preparation for injection, the cells were trypsinized and suspended in Mg+/Ca+ Dulbecco's Phosphate Buffered Saline (DPBS), at a concentration of 1.2×10^5^ cells/μl.

### Animal model

All animal-based procedures were conducted in accordance with the Canadian Council on Animal Care Guidelines. Female immunodeficient Swiss Nude mice (Taconic Farms, Inc. Canada) were used in this study. A total volume of 50 μl of prepared cell suspension was injected subcutaneously into the right hind leg of each mouse, using a 27 gauge needle. Tumors (~8 mm diameter) formed 4-6 weeks post injection. Tumors were permitted to grow to a maximum diameter of 8-10 mm prior to experiments.

Prior to treatment, mice were anesthetised using oxygen ventilated isoflurane for induction and then injected using a mixture of ketamine (100 mg/kg), xylazine (5 mg/kg) and acepromazine (1mg/kg), administered intraperitoneally (I.P.). Anesthetised mice were visually monitored and placed under heat lamps and/or over warmed pads to maintain regular body temperature and limit vasoconstriction due to hypothermia during treatment. Mice observed to experience irregular respiratory rates were administered oxygen.

### Experimental design

Two microbubble concentrations, nil (0%) and high (3% v/v), and three radiation doses, 0 Gy, 2 Gy and 8 Gy were investigated and compared to the control group (no ultrasound, no microbubbles, no radiation). Animals were also investigated in short term (12 h and 24 h) and long term cohorts ( < 28 days). Treatment conditions were applied to the long term and short term animal cohorts and a total of 120 mice were used in this study.

Definity^®^ perflutren ultrasound contrast agents (Lantheus Medical Imaging, Inc., North Billerica, MA, USA) were activated by shaking using a Vialmix^®^ (Lantheus Medical Imaging, Inc., North Billerica, MA, USA) device for 45 seconds at 3000 rpm.

Mice treated with combined ultrasound and microbubble treatments were fitted with a 26 gauge tail vein catheter to facilitate intravenous injection of microbubbles. Each mouse was placed on a custom built mounting device and injected with microbubble suspension followed by 150 μl 0.2% heparin/saline flush. Mice were then partially submerged in a 37.5°C degassed water bath and the tumor-bearing limb was exposed to ultrasound immediately after injection using a custom ultrasound therapy unit. Mice were positioned such that the tumor was placed within the full width half maximum peak of the acoustic signal.

For ultrasound exposures, a focused central frequency 500 kHz transducer (IL0509HP, ValpeyFisher Inc, MA) with a 28.6 mm element diameter was used. A sinusoidal wave was generated by a waveform generator (AWG520, Tektronix, OR), connected to a pulse-receive power amplifier (RPR4000, Ritec Inc, RI) and a digital-acquisition system (Acquiris CC103, Agiulent Technologies NY). Tumors were exposed for 50 milliseconds to a 16 cycle tone burst at 500 kHz with a 3 kHz pulse repetition frequency and a 10% duty cycle. This acoustic burst was repeated every 2 seconds permitting blood vessels to refill with microbubbles. The total treatment lasted for 5 minutes with a total ultrasound exposure time of 750 milliseconds and an average duty cycle of 0.25%. The ultrasound peak negative pressure was 570 kPa measured with a calibrated hydrophone. The −6 dB beam width was 31 mm and the −3 dB beam width was 18 mm. The resulting mechanical index was 0.8; similar to that found in clinical use [43].

### Radiotherapy

Tumors treated with radiation were administered with 0 Gy, 2 Gy, and 8 Gy, at a dose rate of 200 cGy/min, source-to-skin distance (SSD) of 30 cm, and 160 kVP energy, using a Faxitron Cabinet X-ray (Faxitron X-ray, Lincolnshire, IL, USA). To ensure only the tumor was irradiated, a 3 mm thick lead sheet was used with a circular aperture to only expose the tumor to radiation. Radiation was delivered immediately following ultrasound- microbubble therapy.

### Histology

Mice were euthanized by cervical dislocation and tumors subsequently excised at experimental endpoints. Tumor specimens were dissected in half and fixed in 10% neutral-buffered formalin at room temperature for 24 hours. After fixation specimens were embedded in paraffin blocks, sectioned into 5 μm thick slices, mounted on a glass slide, and stained with hematoxylin and eosin (H&E). Samples were also stained using *in situ* end-labelling (ISEL), cluster of differentiation 31 (CD31) and Factor VIII (Pathology Research Program, University Health Network, Toronto, ON, Canada).

Staining with ISEL was used to identify apoptotic cells by internucleosomal DNA fragmentation [44]. Stained histology sections were digitized and the areas of cell death and the average apoptotic index were quantified using ImageJ software (NIH, Bethesda, MD, USA). H&E staining was used to assess cellularity and as a second validation assay for cell death.

Cluster of Differentiation-31 (CD31) targets PECAM-1 adhesion a molecule found on endothelial cells, and was used as a marker for microvessel endothelium. The vascular index was determined by quantifying the number of intact and disrupted vessels manually using a Leica CD100 microscope (20x objective lens, 1MPixel Leica DC100 video camera, 2 GHz PC operating Leica IM1000 software) (Leica GmbH, Germany). Vasculature was quantified for the entire cross sectional area of one tumor slice per animal tumor. The vascular index was calculated as the ratio of the sum of intact luminal vessel number to the total vessel number measured within a region of interest. In order to determine if cell death was a result of vascular damage, Factor VIII was used to identify areas of vascular leakage. Factor VIII is a blood coagulating factor that is activated through a cascade initialized by distressed endothelial cells. Areas of vascular leakage were quantified and normalized per unit area (counts/um^2^).

### Tumor growth

The animals in the long term cohort were treated and followed for up to 28 days. The length (L), height (H) and width (W) of tumors were measured every 2-3 days using a digital calliper. The tumor volume (V) was calculated using VT = [L×H×W×π]/6. Each tumor measurement was normalised by its starting volume (at day 0) and the change in tumor volume (mm^3^) was recorded. Ki- 67 staining was conducted on the tumor samples at end point to identify the growth fraction of the cell population. Expression of Ki-67 protein in the nucleus is present only in active phases of the cell. The Ki-67 labelling index was determined and compared for all treatment groups from the long term cohort.

### Statistics

A two way ANOVA statistical test was performed using GraphPad Prism (GraphPad Software Inc, La Jolla, USA). Significant P-values were indicated between group means and interactions for: radiation dosages (P_mc_), and microbubble-radiation interactions (P_i_).

## ABBREVIATIONS

MB: Microbubble
H&E: hematoxylin and eosin
ISEL: *in situ* end-labelling
CD31: cluster of differentiation 31
