# Erratum: Breast tumor response to ultrasound mediated excitation of microbubbles and radiation therapy in vivo

**DOI:** 10.18632/oncoscience.336

**Published:** 2017-01-30

**Authors:** Priscilla Lai, Christine Tarapacki, William T. Tran, Ahmed El Kaffas, Justin Lee, Clinton Hupple, Sarah Iradji, Anoja Giles, Azza Al-Mahrouki, Gregory J. Czarnota

***Oncoscience. 2016; 3(3-4)*:** 98-108.

**PMCID:** PMC4872648 **PMID:** 27226983

**DOI**: 10.18632/oncoscience.299

The authors amend the acknowledgment section as follows:

